# Dental Health in Smokers with and without COPD

**DOI:** 10.1371/journal.pone.0059492

**Published:** 2013-03-27

**Authors:** Jan Bergström, Kerstin Cederlund, Barbro Dahlén, Ann-Sofie Lantz, Maria Skedinger, Lena Palmberg, Britt-Marie Sundblad, Kjell Larsson

**Affiliations:** 1 Department of Dental Medicine, Karolinska Institutet, Stockholm, Sweden; 2 Department of Clinical Science, Intervention and Technology, Karolinska Institutet, Division of Medical Imaging and Technology, Stockholm, Sweden; 3 Lung and Allergy Research, Karolinska University Hospital Huddinge, Stockholm, Sweden; 4 Lung and Allergy Research, Institute of Environmental Medicine, Karolinska Institutet, Stockholm, Sweden; University of Toronto, Canada

## Abstract

The association between chronic obstructive pulmonary disease (COPD) and periodontal disease is sparsely studied. The aim was to describe the co-variation of periodontitis and lung function impairment in smokers. The hypothesis was that the destructive processes in the mouth and the lungs are interdependent due to a general individual susceptibility to detrimental effects of tobacco smoke. Smokers with COPD (n = 28) stage II and III according to GOLD guidelines and smokers without COPD (n = 29) and healthy non-smokers (n = 23) participated in the study. The groups of smokers were matched for cumulative exposure to tobacco smoke. Radiographic, general and dental clinical examination, lung function measurements and quality of life (SF-36) assessment were conducted. The relationship between respiratory and dental outcomes was analyzed. Dental health, assessed by plaque, gingival bleeding, periodontal pocket depth and loss of teeth was impaired in the smokers compared with non-smokers with no major differences between smokers with and without COPD. There was, however, a weak correlation between periodontitis and emphysema/impaired diffusion capacity. Impaired quality of life was associated with smoking and impaired lung function but not influenced by dental status. In conclusion periodontitis was strongly associated with smoking, weakly associated with lung tissue destruction and very weakly or even not at all associated with chronic airflow limitation. The results indicate that, although there was a co-variation between periodontitis and pathologic lung processes in smokers, the risk of developing COPD, as defined by spirometric outcomes, is not associated with the risk of impaired dental health in smokers.

## Introduction

Periodontal disease and chronic obstructive pulmonary disease (COPD) are characterized by chronic inflammation. The two conditions have a number of features in common such as a chronic trajectory, progressive and irreversible tissue destruction and gradual loss of normal organ function. Both conditions are strongly associated with tobacco smoking. To date there are a few studies in which possible interrelationship between these two conditions has been explored and the results are inconclusive [1]–[7]. Previous observations are based on retrospective studies that were not primarily designed to explore possible associations between COPD and periodontal disease. Thus, despite a clinical impression of an association between dental health and COPD, the scientific evidence for such an association is poor [8].

It is beyond all doubt that smoking is an important risk factor for development of both periodontal disease and COPD and that the retrospectively reported association between the two conditions most likely reflects exposure to tobacco smoke [4]. It is, however, not clear whether the susceptibility to smoke-induced tissue destruction is a general characteristic within an individual or if different tissues within one and the same person react differently to the harmful effects of smoking. If there is a general susceptibility to the harmful effects of tobacco smoke the development of periodontal disease and chronic airflow limitation would be associated and there would be a co-variation between the destructive processes in the mouth and the lungs. If the sensitivity to tobacco smoke is not a general individual characteristic destructive processes in the mouth will develop independently of alterations in the lungs.

The present study was carried out with the aim to investigate the dental health condition in terms of dental plaque, gingival inflammation, periodontal pocket depth, and tooth loss in two groups of smokers with similar cumulative exposure to tobacco smoke, one group with a confirmed diagnosis of COPD and another group that did not fulfil the criteria for COPD. For comparison a control group of healthy non-smokers was included. We hypothesized that the susceptibility to smoking induced tissue damage is a general characteristic within an individual and that there is an association between the development of tissue destruction in the lungs and in the mouth.

## Material and Methods

### Study Population and Study Design

Participants were recruited by advertisement in the daily press and from the clinic. Current smokers who denied heart disease or other severe diseases were eligible for inclusion. Smokers with a post-bronchodilator FEV_1/_FVC <0.7 and FEV_1_ 40% to 70% of predicted value constituted the COPD group. Smokers with a post-bronchodilator FEV_1_/FVC >0.70 and a FEV_1_>70% of predicted value constituted the non-COPD group. The control group included healthy, never-smokers with normal spirometry and no history of asthma, allergy or other pulmonary diseases.

Eighty individuals (40 women), 28 smokers with COPD, 29 smokers without COPD and 23 healthy non-smokers fulfilled the inclusion criteria (table 1).

**Table 1:** Baseline characteristics of the participants.

	Non-smokers (n = 23)	Smokers without COPD (n = 29)	Smokers with COPD (n = 28)
Gender, female/male	8/15	15/14	17/11
Age, year (range)	55 (41–72)	53 (38–66)	61 (48–73)** ###
BMI, kg/m^2^	25.0 (23.7–26.4)	25.1 (23.9–26.3)	23.7 (22.5–24.8)
Smoking, pack year	–	35.6 (27.5–43.7)	37.3 (33.8–40.9)

Data are given as mean values (range) or (95% confidence intervals).

** indicates p<0.01 compared with non-smokers.

### indicates p<0.001 compared with smokers without COPD.

After clinical examination all participants underwent measurement of lung function and exhaled NO and completed a quality of life questionnaire (Short formula 36, SF-36). Subjects with COPD also completed the S: t Georges Respiratory Questionnaire (SGRQ). On a separate day pulmonary X-ray and computerized tomography (CT) of the thorax were performed. On a third day a dental examination was conducted.

### Ethics Statement

The participants were informed (verbally and in writing) about the purpose of the study and signed an informed consent. The study was approved by the regional Ethic Committee in Stockholm (Dnr 2005/733 31/1-4).

### Lung Function

Lung function (vital capacity, VC, forced vital capacity, FVC, forced expiratory volume in one second, FEV_1_, residual volume, RV, functional residual capacity, FRC, and total lung capacity, TLC) was measured according to the American Thoracic Society criteria [9], [10] using body box (Jaeger®, Würtsberg, Germany). Local reference values were used [11], [12]. Single breath diffusion capacity (D*l*_CO_) was measured using single breath carbon monoxide technique [10].

Exhaled levels of nitric oxide (NO) was measured (Niox, Aerocrine, Solna, Sweden) according to ATS criteria [13].

### Chest X-ray

Chest images were obtained by computed radiography technique with phosphor storage plates (Fujifilm DR Velocity Ufp; Fujifilm Corporation, Tokyo, Japan) with exposure factors similar to those used in clinical practice (150 kV, automatic exposure control). The images were transferred to the PACS (picture, archiving and communicating system) of the radiology department for soft copy viewing.

Two senior thoracic radiologists in consensus performed semi-quantitative evaluation of emphysema and bronchial wall thickness. Signs of emphysema were defined as low-attenuation areas, altered vascular pattern (e.g. thin and sparse vessels with few branches) or a combination of these. Emphysema and bronchial wall thickness was separately graded on a 4-point scale (0 = normal, 1 = slight, 2 = moderate and 3 = pronounced). The PA-image was divided into 4 squares (right upper, right lower, left upper and left lower) and each square was separately graded. Thus, a maximum score of either emphysema or bronchial wall thickness for a PA-image was 12.

### Computed Tomography(CT) Acquisition

All patients underwent a 64-slice helical CT examination of the chest (Siemens Sensation 64, Siemens company, Erlangen, Germany) using the following parameters: collimation 64×0.625 mm, rotation time 0,5 sec, pitch 1 and 120 kV. Images were reconstructed with a high frequency convolution kernel of 1.2 and axial slice thickness of 5 mm.

Two senior thoracic radiologists in consensus performed visually, semi quantitative evaluation of emphysema and bronchial wall thickness using a 4-point scale (0 = normal, 1 = slight, 2 = moderate and 3 = pronounced) for the 5 lung lobes separately (left upper, left lower, right upper, right lower and the middle lobe). Thus, a maximum score of either emphysema or bronchial wall thickness was 15.

### Dental Examination

The dental examination focused on periodontal health condition and included assessments of remaining teeth, periodontal pocket depth, gingival inflammation, gingival recession, and dental plaque as a measure of the oral hygiene standard. Pocket depth was measured with a millimetre graduated probe with a special marking at the 4 mm level (Hu-Friedy PC PUNC 15) at four sites per tooth (mesio-buccal, mesio-lingual, disto-buccal and disto-lingual). Sites with a pocket depth of 4 mm or more were measured to the nearest mm. Sites with a depth of less than 4 mm (non-diseased or healthy sites) were set to 2 mm. The mean pocket depth was calculated based on all remaining teeth, third molars excluded. If the first or second molar was missing it was replaced by the third molar in the same quadrant if normally erupted. Pocket depth of missing tooth sites was set to the values of neighbouring tooth sites. In a small number of individuals (n = 6) who obviously had lost teeth due to periodontal disease the pocket depth was set to 9 mm. In these 6 patients analyses of medical records from previous care givers confirmed the relationship between loss of teeth and periodontitis. A mean pocket depth of ≥4 mm was arbitrarily chosen as cut off point to define periodontal disease.

Estimation of gingival inflammation severity was based on gingival bleeding propensity upon probing in conjunction with pocket depth measurement. Gingival bleeding was dichotomously assessed as present or not within 30 seconds following probing with gentle pressure at four sites per tooth and expressed as a percentage of bleeding positive surfaces. Gingival recession was measured as the distance from the gingival margin to the cement-enamel junction (or to a corresponding point where this anatomic landmark was obscured) and given as the percentage of teeth with one or more sites exhibiting recession.

The occurrence of dental plaque was dichotomously assessed following staining with Erythrosine (Diaplack Pellets, TM) on four supra-gingival surfaces per tooth and expressed as a percentage of plaque positive surfaces.

### Quality of Life

In all three groups quality of life was assessed by the Swedish SF-36 Health Survey [14] with eight dimension scales. Mental (MCS) and physical (PCS) component summary scores were calculated.

The COPD group completed the COPD specific St. Georgés Respiratory Questionnaire (SGRQ) containing three domains: symptoms, activity and impact [15]. Scores from each domain and total score were assessed.

### Statistics

Results were expressed as means and 95% confidence intervals (95% CI). Group means for dental plaque, gingival bleeding, and pocket depth were based on intra-individual means (case means). Statistical analysis was performed using multifactorial analysis of variance, including *post hoc* multiple comparison testing according to Scheffé. Variables based on categorical data were tested following the Chi-square distribution with Yates correction of continuity. Multiple linear regression analysis was conducted with pocket depth as the dependent variable and age, gender, dental plaque, gingival bleeding, chronic airflow limitation (yes/no), and smoking (yes/no) as explanatory variables. Relative risk estimation was based on logistic regression with mean pocket depth (dichotomized, <or ≥4 mm) as dependent variable, and age, gingival bleeding, dental plaque, COPD (yes/no), and smoking (yes/no) served as independent variables. Subgroup analyses of possible relationship between periodontal and lung alterations were assessed by non-parametrical Spearmans rank correlation. The STATISTICA software program version 9.1 (Statsoft Scandinavia AB, Uppsala Sweden) was used for the statistical analysis. A p-value of <0.05 (two-sided) was considered statistically significant.

## Results

### Lung Function and Chest Imaging

The cumulative exposure to tobacco smoke was similar in the two groups of smokers. Smokers with COPD were somewhat older than the other two groups. Apart from a low diffusion capacity lung function did not differ between smokers without COPD and non-smokers (table 2). Exhaled NO levels were lower in the two groups of smokers than in the non-smokers but did not differ between the two groups of smokers (table 2).

**Table 2:** Lung function and chest imaging in the non-smokers, smokers without and smokers with COPD.

	Non-smokers (n = 23)	Smokers without COPD (n = 29)	Smokers with COPD (n = 28)	P
**Lung volumes**				
TLC, % pred	110 (102–118)	110 (105–115)	120 (115–126)**^#^	0.003
VC, % pred	99 (93–104)	96 (91–100)	82 (76–87)* ^##^	0.003
RV, % pred	146 (136–156)	139 (131–148)	192 (177–206)***^###^	<0.001
FRC, % pred	113 (105–121)	113 (106–119)	135 (126–145)***^###^	<0.001
**Dynamic spirometry**				
FVC, % pred After bronchodilatation	98 (93–102)	95 (90–100)	80 (73–86)***^###^	<0.001
FEV_1_, % pred After bronchodilatation	102 (97–106)	96 (91–100)	57 (50–63)***^###^	<0.001
FEV_1_/FVC After bronchodilatation	0.80 (0.77–0.82)	0.77 (0.74–0.79)	0.54 (0.51–0.58)***^###^	<0.001
**D** ***l*** **_CO_ and exhaled NO**				
D*l*_CO_, % pred	91 (85–98)	73 (68–78)***	48 (42–54)***^###^	<0.001
NO, ppb	23.6 (14.7–32.5)	13.9 (11.6–16.1)*	12.8 (10.2–15.5)**	0.004
**Imaging**				
CXR emphysema	1.6 (0.73–2.6)	2.4 (1.4–3.5)	5.8 (4.9–6.8)***^###^	<0.001
CXR bronch wall	0.7 (0.2–1.3)	2.1 (1.3–2.9)*	3.3 (2.7–3.9)***^#^	<0.001
CT emphysema	0.3 (−0.2–0.8)	2.2 (1.2–3.1)*	7.5 (6.1–8.9)***^###^	<0.001
CT bronch wall	1.6 (0.4–2.8)	3.2 (2.2–4.1)	6.6 (5.2–8.0)***^###^	<0.001

Data are given as mean values (95% confidence interval).

TLC: total lung capacity, VC: vital capacity, RV: residual vloume, FRC: functional residual capacity, FVC: forced vital capacity, FEV_1_: forced expiratory volume in one second, D*l*_CO_: diffusion capacity, NO: nitric oxide, CXR: chest X-ray, CT: computerized tomography, Bronch wall: thickness of the bronchial wall.

* , **, ***indicate p<0.05, p<0.01 and p<0.001, respectively, compared with non-smokers.

#, ##, ### indicate p<0.05, p<0.01 and p<0.001, respectively, compared with smokers without COPD.

Bronchial wall thickness and emphysema score as assessed by chest X-ray and CT were increased in the COPD group compared with the other two groups (table 2). There were also minor, for some outcome variables significant, differences between the smokers without COPD and the non-smokers in this respect (table 2).

### Dental Status

**Figure 1:**
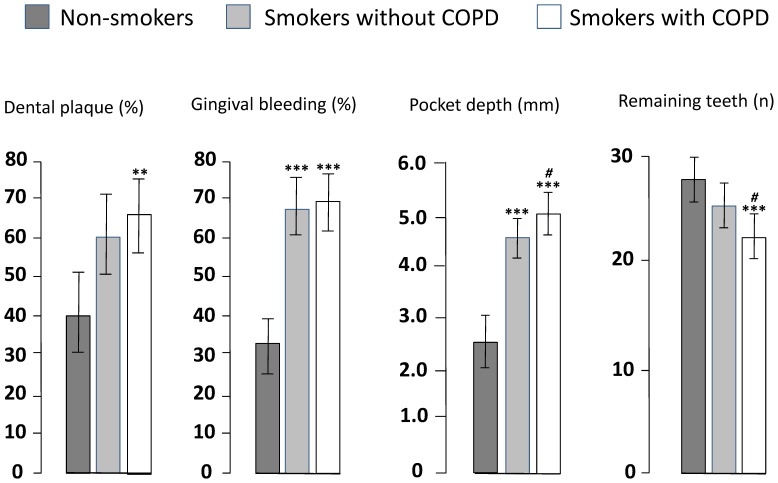
Dental plaque, gingival bleeding, pocket depth and remaining teeth in 28 smokers with COPD, 29 smokers who did not fulfill the criteria for COPD, matched for smoking, and 23 healthy non-smokers. Mean values and 95% confidence intervals. There is an overall difference between the groups for all outcomes (dental plaque p<0.01, gingival bleeding p<0.001, pocket depth p<0.0001, remaining teeth p<0.001). **and ***indicate p<0.01 and p<0.001, respectively compared with non-smokers. ^#^ indicates p<0.05 compared with smokers without COPD.

Dental plaque, gingival bleeding and pocket depth were increased in both groups of smokers compared with non-smokers. The number of remaining teeth was reduced in the COPD group compared with the other two groups. Smokers with COPD had somewhat inferior dental status than smokers without COPD, the differences being significant for pocket depth (p<0.01) and remaining teeth (p<0.05; figure 1).

Dental plaque was significantly more prevalent in the two groups of smokers than in non-smokers when controlled for age and gender (p<0.01). When controlled for age, gender and dental plaque both number of sites exhibiting gingival bleeding and pocket depth was elevated in both groups of smokers in comparison with non-smoking controls (p<0.0001 for both), the latter also being increased in the COPD group compared with smokers without COPD (p<0.01). The mean number of remaining teeth was significantly reduced in smokers with COPD compared with non-smokers (p<0.001) and smokers without COPD (p<0.05) controlling for age and gender (figure 1). The mean (95% CI) percentage of teeth exhibiting gingival recession was 37.7 (22.2–50.7), 29.7 (15.0–39.6), and 8.9 (2.3–15.5) in smokers with COPD, smokers without COPD, and non-smokers, respectively. The difference was statistically significant between COPD and non-smokers controlling for age and gender (p<0.01).

Multiple regression analysis was conducted with mean pocket depth as the dependent variable and age, gender, dental plaque, gingival bleeding, COPD-status (yes/no) and smoking (yes/no) as explanatory variables. The analysis resulted in a significant model (F = 25.2, p<0.0001, R^2^_adj = _0.66). Smoking, dental plaque, and gingival bleeding were statistically significant explanatory variables (p<0.05 for all) whereas COPD and age were not.

Logistic regression with mean pocket depth as a dichotomous variable (≥4.0 mm = 1, <4.0 mm = 0) and age, gingival bleeding, dental plaque, COPD-status, and smoking as independent variables resulted in a significant model (Chi^2^ = 50.9, p<0.0001). Smoking was the only statistically significant factor indicating a considerable smoking associated over-risk for the presence of periodontal pockets in this population (OR = 24.2, 95% CI 2.0–286.8, p<0.01). Using this definition of periodontal disease the prevalence was 82% for smokers with COPD, 58% for smokers without COPD, and 4% for non-smokers.

Subgroup analyses of the two groups of smokers revealed weak but significant correlations between occurrence of emphysema, as assessed by evaluation of CT-examination, and pocket depth (Rho = 0.29; p = 0.039) as well as between emphysema and teeth loss (Rho = −0.28; p = 0.043). Also, pooled data from the two groups of smokers showed a correlation between diffusion capacity (DLCO) and teeth loss (Rho = 0.40; p = 0.004).

### Quality of Life

**Figure 2:**
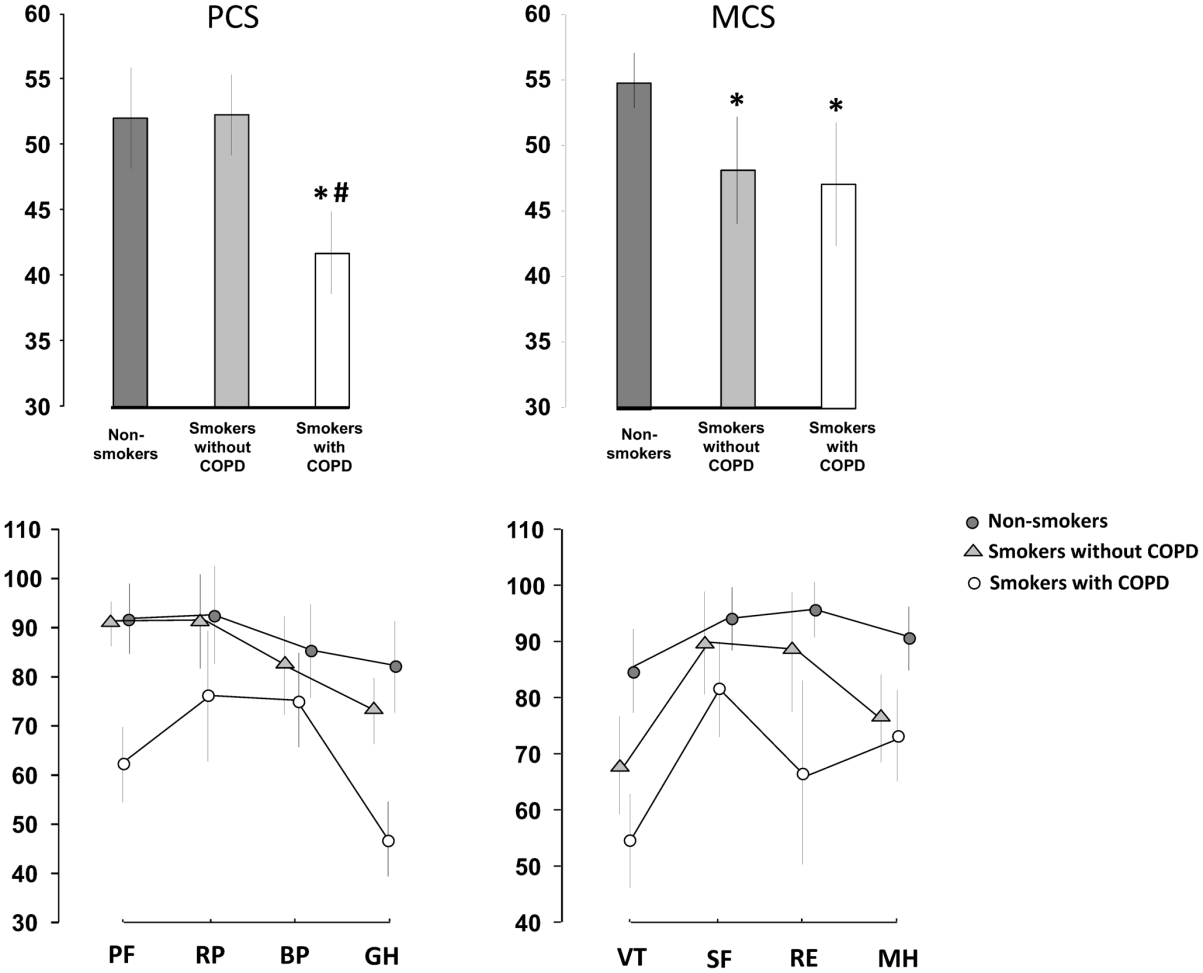
Quality of life assessed by the general quality of life instrument SF-36 in 28 smokers with COPD, 29 smokers with no airflow limitation and 23 healthy non-smokers. PCS: physical component summary, MCS: mental component summary, PF: physical functioning, RP: role-physical, BP: bodily pain, GH: general health, VT: vitality, SF: social functioning, RE: role emotional, MH: mental health. Data are presented as mean values and 95% confidence intervals. *indicates p<0.001 compared with non-smokers. ^#^indicates p<0.001 compared with smokers without COPD.

The quality of life as assessed by the SF-36 revealed lower physical component summary (PCS) in smokers with COPD (42 (39–45)) than in smokers without COPD (52 (49–55); p<0.001) and non-smokers (52 (48–56); p<0.001) with no significant difference between the smoking groups. In particular, the COPD group had low physical functioning (PF) scores and the general health (GH) scales (figure 2).

The SF-36 mental component summary (MCS) was 47 (42–52) in the COPD group and 48 (44–52) in the smokers without COPD which was lower than in the non-smokers (55 (53–57); p<0.05 for both). No difference was observed between the two groups of smokers. The two groups of smokers had low scores on vitality (VT) and mental health (MH) scales whereas smokers with COPD also had low scores on the role emotional (RE) scale (figure 2).

Assessed by SGRQ the quality of life total score was 40 (34–47), the symptom domain 58 (48–68), the activity domain 53 (45–61) and the impact domain 29 (22–36) in the COPD group.

There was in general poor correlation (coefficients of correlation mostly between 0.20 and 0.25) between dental outcome measures and quality of life assessed as PCS and MCS when correction was made for age, gender and lung function.

## Discussion

In the present study it was demonstrated that smokers with and without COPD, with similar cumulative exposure to tobacco smoke, have a severely impaired dental status. It was found that the detrimental impact on dental health in smokers is primarily associated with the exposure and not with development of chronic airflow limitation. Thus, susceptibility to the harmful effects of smoking is not a general characteristic within an individual smoker, destructive effects in the mouth occurred independent of development of COPD. Our results clearly showed that most smokers are stricken with pathological alterations in the oral cavity whereas the development of chronic airflow limitation is restricted to a subgroup of smokers. This supports the idea that the mechanisms behind smoking induced tissue damage in the mouth and the lungs differ, likely reflecting inter-individual variation in organ-related susceptibility to the harmful effects. This is also supported by the finding of major radiographic (emphysema) and physiologic (diffusion capacity) differences between the two groups of smokers whereas the differences in dental status were minor.

There was a slight tendency towards a worse dental status in the smokers who developed chronic airflow limitation than in smokers with normal spirometry. Thus, pocket depth was increased and the number of remaining teeth reduced in the smokers with COPD compared with the smokers who did not have chronic airflow limitation. This difference was minor and is likely due to the difference in age between the groups. Thus, when age was controlled for in the multivariate statistical models, chronic airflow limitation was not associated with increased risk for any of the outcome measures of dental status. As our groups of smokers consist of just below 30 individuals in each group, it may be difficult to draw firm conclusions about the lack of difference between the two groups of smokers. Despite the rather small groups, we found a weak, but statistically significant, association between periodontal disease and lung pathology indicating a true co-variation between periodontal alterations, such as pocket depth and teeth loss, and pathologic lung processes such as emphysema and impaired diffusion capacity. This indicate that there may be a relationship between destructive processes in the oral cavity and the lungs which are not reflected by changes in lung function assessed by spirometry and thus not related to the development of COPD according to current definition of the disease.

The relationship between dental health and COPD has not been much studied. An association between COPD and periodontal disease was suggested in an epidemiologic study. In that study, however, no lung function data were reported making firm conclusion about the COPD diagnosis difficult [5]. Furthermore, that study was based on smokers and non-smokers above the age of 20 years, thus including a substantial number of subjects who probably were too young to have developed COPD. In a study of radiographic examination of dental status (orthopantomogram) it was found that periodontal disease was more common in patients with very severe COPD than in severely ill patients evaluated for lung transplantation due to other severe respiratory diseases [4]. There was a considerable difference in smoking history between the groups but the difference in dental status remained after correction for smoking habits in the group with very severe COPD [4]. In a study by Hyman and Reid a relationship between periodontal disease and smoking was established but no relationship between periodontal disease and COPD [2]. Also Katancik et al found an association between poor dental status and impaired lung function [3] that, after correction for smoking only appeared in former smokers.

Fewer remaining teeth were found in the COPD group compared to the other two groups also after control for age. This may, to a minor extent, be explained by the fact that periodontal disease was a little more advanced in smokers with COPD than in non-COPD smokers and, as a consequence, the risk for loss of teeth increased in that group. However, tooth loss is not a specific measure of periodontal disease but is equally often a result of dental caries, which was not assessed in the present study. The finding is, to some extent, supported by those of Wang et al [7] who found fewer remaining teeth in patients with COPD than in a control group. In that study, however, one third of the COPD patients were non-smokers and one third in the control group was current or former smokers making conclusion about the causality between smoking, COPD and tooth loss difficult.

In the present study pocket depth was the main determinant of periodontal disease. In other studies attachment loss has been used for this purpose [1], [2]. The most commonly used periodontal disease variable, pocket depth, does not, unlike attachment loss, account for possible recession of the gingival margin. We therefore assessed gingival recession as a separate variable. We found that gingival recession was more prevalent among smokers (with and without COPD) than in non-smokers, possibly suggesting that measurement of pocket depth may underestimate the disease severity in the two groups of smokers. Further, we used a standardized pocket depth value of 2 mm for (healthy) sites that did not reach a depth of 4 mm (critical level of disease). This may to some extent underestimate the condition of healthy sites in persons with multiple diseased sites. These limitations suggest that current data, as presented, may be slightly conservative but did not, however, influence the main outcome of the study.

The purpose for quality of life assessment was to find out whether or not our patients with COPD stage II and III had an impaired quality of life and, if so, to what extent an altered dental status contributes to this impairment. In general, the COPD patients in the present study had moderate or severe disease, stage II or III according to GOLD criteria [16] with impaired quality of life as assessed by the disease specific SGRQ with an average total score of 40. As expected, this is slightly better, than the quality of life recently found in two large COPD trials including more than 12000 patients [17], [18]. Compared with the present results, the patients in these studies had a more severe disease; in the present study most of the patients were in stage II, i e had a FEV_1_>50% of predicted value, whereas FEV_1_, on average, was below 50% of predicted value indicating that most of the patients were in stage III according to GOLD criteria in the previous two studies.

In the COPD group poor quality of life was observed in the symptom domain which is in agreement with the low scores in the SF-36 physical performance scales. Thus, total score for the physical component in the SF-36 and, in particular physical functioning and general health scales, were impaired in the COPD group while the smokers without COPD did not differ from non-smokers in this respect. In contrast to the physical component of SF-36 the mental component was lower in both groups of smokers than in non-smokers, the differences being most obvious in the vitality and mental health scales. We conclude that, while impaired physical component summary relates to impaired lung function and not to smoking *per se*, impaired mental component summary was more related to smoking than to the presence of chronic airflow limitation. The reason for this is unclear. Furthermore, the results clearly indicate that dental status had only a minor influence on quality of life as assessed by the SF-36.

Our COPD patients had a slightly better quality of life with regard to the physical component, whereas the mental component was similar as assessed by a generic instrument (SF-36) when compared with stage II and III COPD patients in a recently published study [19]. From these previous assessments of general and disease specific quality of life we conclude that the patients in the present study were representative for COPD patients in GOLD stage II and III.

In conclusion, smoking induces chronic airflow limitation in some smokers who possess a specific susceptibility to tobacco smoke whereas smoking seems to cause periodontal disease in a larger portion of the smokers. Although pathologic processes in the mouth cavity weakly co-varied with pathological processes in the lungs (emphysema and impaired diffusion capacity), we conclude that the susceptibility to the harmful effects of smoking is not a general characteristic within an individual smoker as the association between chronic airflow limitation and periodontitis tangibly varied among smokers.
